# Fc-Epsilon-RI, the High Affinity IgE-Receptor, Is Robustly Expressed in the Upper Gastrointestinal Tract and Modulated by Mucosal Inflammation

**DOI:** 10.1371/journal.pone.0042066

**Published:** 2012-07-27

**Authors:** Christina Bannert, Bettina Bidmon-Fliegenschnee, Georg Stary, Florian Hotzy, Judith Stift, Samuel Nurko, Zsolt Szépfalusi, Edda Fiebiger, Eleonora Dehlink

**Affiliations:** 1 Department of Paediatrics and Adolescent Medicine, Medical University of Vienna, Vienna, Austria; 2 Department of Dermatology, Division of Immunology, Allergy and Infectious Diseases (DIAID), Medical University of Vienna, Vienna, Austria; 3 Clinical Institute of Pathology, Medical University of Vienna, Vienna, Austria; 4 Division of Gastroenterology and Nutrition, Children’s Hospital Boston, Harvard Medical School, Boston, Massachusetts, United States of America; New York University, United States of America

## Abstract

**Background:**

The role of the high affinity IgE receptor, FcεRI, in IgE-mediated immune responses of the gastrointestinal (GI) mucosa is poorly understood. Currently, a detailed characterization of FcεRI expression throughout the human gut is lacking. The aim of this study was to define the expression pattern of FcεRI in the GI tract.

**Methods/Principal Findings:**

We compared FcεRI expression in children with gastritis/esophagitis (n = 10), celiac disease (n = 10), inflammatory bowel disease (IBD) (n = 9), and normal mucosa (n = 5). The α–subunit of FcεRI (FcεRIα), detected by immunohistochemistry, was found on cells infiltrating the mucosa of the esophagus, the stomach, and the duodenum, but was rarely detected in more distal sections of the GI tract. Accordingly, quantitative RT-PCR analysis on esophagus, stomach, duodenum, colon, and rectum biopsies revealed that FcεRIα and -β expression levels decreased towards the distal intestine. mRNA transcripts of the common Fc-receptor-γ chain were present in the entire GI mucosa. Double-immunofluorescence staining of esophageal specimens confirmed that FcεRIα was expressed on intraepithelial mast cells and Langerhans cells. The mRNA expression levels of the α, β, and γ subunits of FcεRI did not correlate with total serum IgE but were associated with mucosal inflammation.

**Conclusion/Significance:**

Our data define the upper GI tract as the main site for IgE-mediated immune activation via FcεRI. Tissue mRNA levels of FcεRIα are regulated by inflammatory conditions rather than serum IgE, indicating that FcεRI might also play a role in pathologies other than allergy.

## Introduction

The gastrointestinal (GI) mucosa is a large interface area for pathogens and environmental antigens and, therefore, is under constant surveillance of the immune system. Immunoglobulin (Ig) receptors are gatekeepers of host defense at mucosal surfaces; they shuttle Ig-antigen complexes across the healthy epithelium and activate protective immune responses. Misguided immune responses, however, can lead to inflammation of the gut or other allergic reactions towards harmless allergens. IgE and its cellular receptors are key players in allergic reactions and parasite defense. Humans express three IgE-receptors, the high affinity IgE-receptor, FcεRI, and two low-affinity IgE receptors, FcεRII (or CD23), and ε-binding protein εBP (or galectin 3) [1]. In the human GI mucosa, the expression of the low affinity IgE receptors is well documented; CD23 is expressed on intestinal epithelial cells and functions as an antigen-sampling protein for IgE-antigen complexes, implying that CD23 plays a role in food allergy [2], [3], [4], [5], [6], [7]. Galectin 3 has been shown to be downregulated during intestinal inflammation and is associated with colon cancer progression [8], [9], [10], [11]. There is little data, however, on the expression profile of the high affinity receptor FcεRI in the gastrointestinal mucosa.

FcεRI is a multimeric receptor of the immunoglobulin receptor superfamily and binds the Fc-part of IgE with its immunoglobulin domain-containing α-chain. Allergen-mediated crosslinking of IgE-FcεRI complexes on the surface of blood and tissue cells then triggers the allergic cascade via the receptor’s signaling subunits, FcεRIβ and FcεRIγ [12]. Human FcεRI is expressed in a tretrameric form (FcεRIαβγ_2_) on the surface of mast cells and basophils, and in a trimeric form (FcεRIαγ_2_) on eosinophils, macrophages, and dendritic cells (DCs) [1]. In peripheral blood, the majority of FcεRI-expressing cells carry IgE [13], [14]. Since binding of IgE to FcεRIα stabilizes the IgE-receptor complex, cell surface expression of FcεRI on peripheral blood cells has been shown to tightly correlate with serum IgE levels as well as cell-bound IgE [15], [16], [17].

In the GI tract, FcεRI-expressing DCs of the Langerhans cell type have been described in the oral mucosa [18] and in the esophageal epithelium of children with gastroesophageal reflux and Eosinophilic Esophagitis (EoE), an allergic condition of the upper GI tract [19]. FcεRI is the only IgE receptor that is expressed in the esophagus [19]. Untersmayr et al. detected FcεRI-positive epithelial cells in the terminal ileum and the colon of colon cancer patients and patients with inflammatory conditions of the gut [20]. Previously, IgE-loaded mast cells have been described in the intestinal mucosa of food allergic- as well as healthy individuals [21], [22].

A detailed analysis of mucosal FcεRI expression throughout the GI tract is currently not available. The aim of the present study, therefore, was to characterize the expression pattern of FcεRI throughout the GI tract and to investigate the impact of serum IgE levels and mucosal inflammation on FcεRI expression levels.

## Results

### Study Population

We investigated mucosal specimens from a total of 34 pediatric patients (15 girls, 19 boys, median age at time of endoscopy 12.4 years). Patients had a diagnosis of gastritis/esophagitis (n = 10), celiac disease (n = 10), or inflammatory bowel disease (IBD) (n = 9). Biopsies of 5 patients did not show any mucosal pathology and served as normal controls. Total serum IgE was measured at the time of endoscopy. Fifteen patients had elevated serum IgE levels (gastritis/reflux n = 5, celiac disease n = 5, IBD n = 4, normal n = 1). In 19 patients, IgE levels were within the normal range. Patients’ characteristics are summarized in Table 1. Children were not routinely tested for the presence of intestinal parasites or helminths, but the expected prevalence for such infections is negligible in a Central European country.

**Table 1:** Patients’ characteristics.

	n	Sex (f/m)	Age at endoscopyMedian (Range)	IgE (kU/l) Median (Range)	z-units Median (Range)	Elevated IgE (n)
**Total**	34	15/19	12.37 (2.72–18.03)	21.90 (2.0–1,510)	0.52 (−1.74–3.81)	15
**Gastritis/Esophagitis**	10	3/7	12.93 (2.72–16.86)	27.55 (2.8–300)	0.55 (−1.74–2.38)	5
**Celiac’s Disease**	10	7/3	8.69 (3.18–17.44)	22.35 (0–630)	0.66 (−1.57–3.81)	5
**IBD**	9	2/7	15.30 (6.15–18.03)	7.5 (0–1,510)	−0.86 (−1.68–3.73)	4
**Normal Mucosa**	5	3/2	11.93 (3.36–12.49)	33.40 (9.4–347)	0.52 (−0.62–2.54)	1

IBD: inflammatory bowel disease.

### Histological Detection of FcεRI in the GI Mucosa

**Figure 1:**
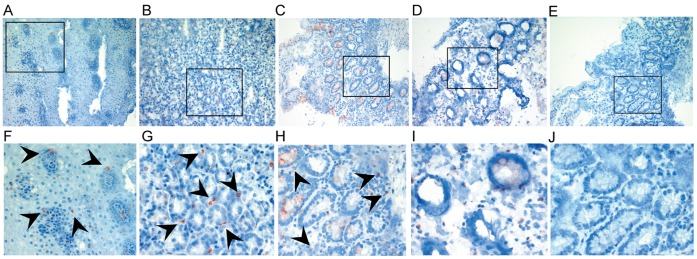
Immunohistochemistry with FcεRIα specific antibody (mAb 15-1) on snap-frozen intestinal specimens from (A) the esophagus, (B) the stomach, (C) the duodenum, and (D) the colon. FcεRIα-positive cells (red) are frequently found in the esophagus, the stomach, and the duodenum (black arrows). (E) shows isotype control with mouse IgG1. Goblet cells in the duodenum and the colon revealed non-specific binding of antibodies. Original magnification x20. Bottom row (F-J) shows details from A-E. Representative specimens from n = 10.

**Figure 2:**
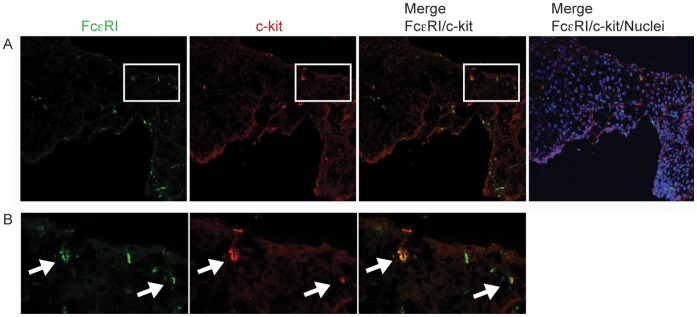
c-kit positive mast cells in the esophagus epithelium express FcεRIα. (A) FcεRIα is visualized with mAb Cra1 (green, first panel). Mast cells are shown with c-kit as a marker (red, second panel). Cell nuclei are visualized with DAPI staining (blue). (B) shows higher magnifications from (A). FcεRIα is expressed on esophageal mast cells (B, white arrows). Representative specimens from n = 3.

**Figure 3:**
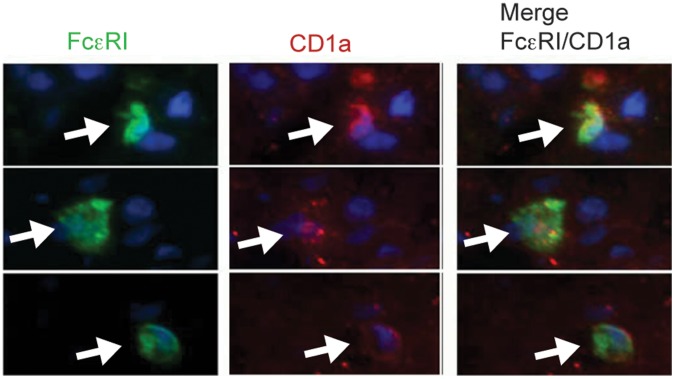
CD1a-positive Langerhans cells in the esophagus epithelium express FcεRIα. FcεRIα is visualized with mAb Cra1 (green, first panel). Dendritic cells are shown with the Langerhans cell marker CD1a (red, second panel). Cell nuclei are visualized with DAPI staining (blue). Representative specimens from n = 3.

We conducted immunohistochemistry staining of snap-frozen mucosal biopsies from all sections of the GI tract accessible by endoscopy. FcεRIα was frequently found on cells infiltrating the mucosa of the upper GI tract (esophagus, Figure 1A and F, stomach Figure 1B and G, and duodenum, Figure 1C and H). Specimens from the lower GI tract (terminal ileum, colon, and rectum) showed only scattered FcεRI positive cells (Figure 1D and I). We recently identified Langerhans cells and mast cells as the main bearers of FcεRI in the esophageal epithelium by immunohistochemistry double-staining [19]. Here, double-immunofluorescence staining of snap-frozen esophageal specimens confirmed that FcεRI was expressed on c-kit-positive intraepithelial mast cells (Figure 2) and CD1a-positive Langerhans cells (Figure 3).

### FcεRIα, ß, and γ are Robustly Expressed in the Upper Gastrointestinal Tract

**Figure 4:**
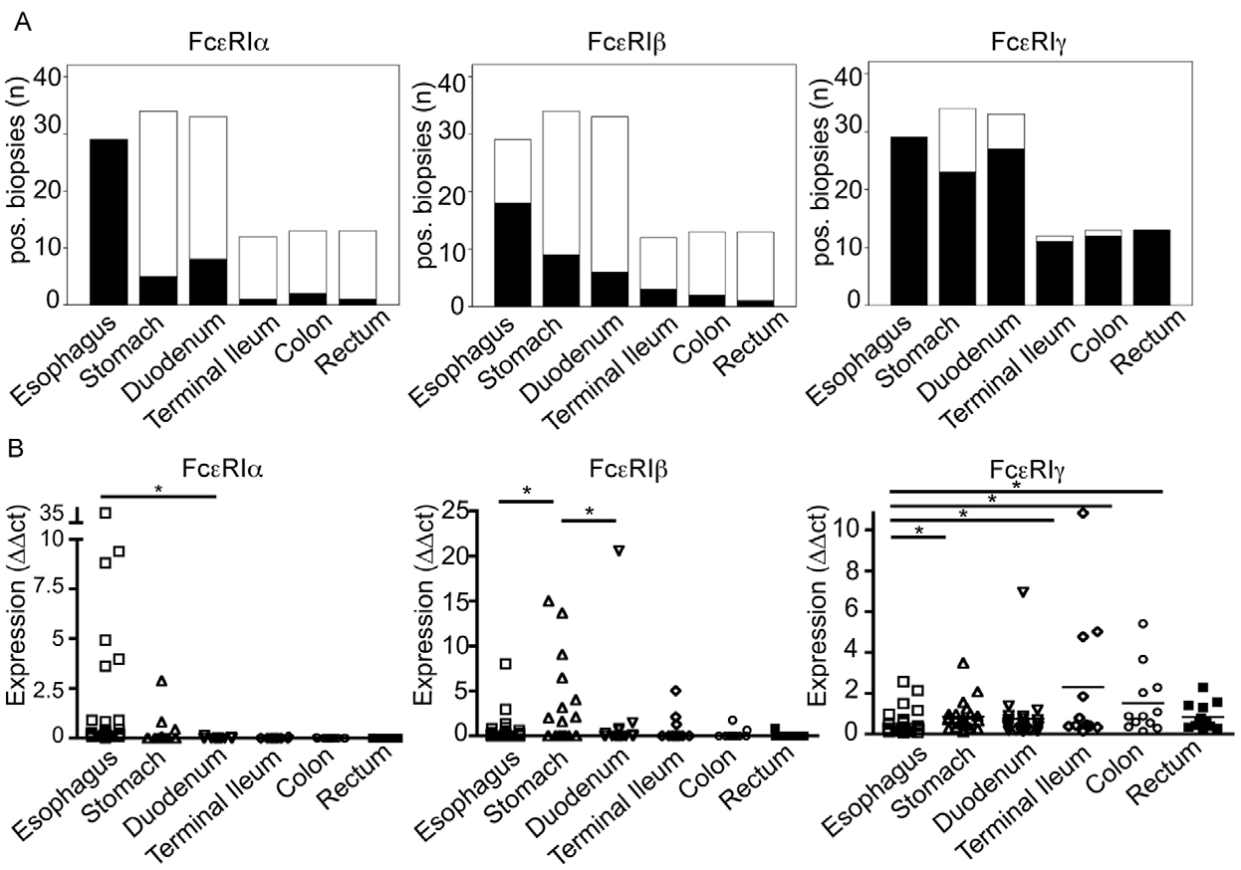
Quantitative RT-PCR for the three subunits of the high affinity IgE-receptor was performed on intestinal biopsies from pediatric patients. (A) FcεRIα mRNA transcripts were found in all esophageal biopsies and with varying frequency in more distal biopsies (left panel). Similarly, the highest frequency of FcεRIβ mRNA-positive specimens was found in the esophagus (middle panel). The common Fc-γ chain was detected in the majority of specimens from the entire GI tract (right panel). Black stacked-bars represent target-positive specimens, and white stacked-bars represent target-negative specimens. (B) The highest levels of FcεRIα mRNA transcripts were found in the esophageal mucosa (left panel), while FcεRIβ mRNA expression peaked in the gastric mucosa (middle panel). Esophageal specimens revealed the lowest FcεRIγ mRNA expression compared to the stomach, terminal ileum, colon, and rectum (right panel). * p<0.05, Mann-Whitney-U test.

Because immunohistochemistry does not allow for quantification of protein expression levels and might not be sensitive enough to detect low-level expression of FcεRI subunits, we employed quantitative RT-PCR to screen gastrointestinal biopsies for FcεRIα-, -ß-, and -γ-mRNA. We detected FcεRIα mRNA in all esophageal biopsies (29/29), in 5/34 gastric samples, 8/33 duodenum samples, 1/12 terminal ileum samples, 2/13 colon samples, and 1/13 rectum samples (Figure 4A, left panel). FcεRIβ transcripts were found in 18/29 esophageal samples, 9/34 stomach samples, 6/33 duodenum samples, 3/12 terminal ileum samples, 2/13 colon samples, and 1/13 rectum samples, Figure 4A, middle panel). In line with its function as a common signaling chain for several Fc receptors [23], Fc-receptor-γ mRNA was present in most specimens from all sections of the gut (right panel). FcεRIα mRNA levels were highest in the esophagus and decreased along the GI tract (Figure 4B, left panel). FcεRIβ mRNA-expression peaked in the gastric mucosa (middle panel). Esophageal specimens were low in FcεRIγ transcripts compared to the stomach, terminal ileum, colon, and rectum (right panel, Mann-Whitney-U test). This data demonstrate that, as measured at the mRNA level, the FcεRIα and β subunits are robustly expressed in the proximal parts of the GI tract and their expression levels decrease towards the distal intestine, while the common Fc-receptor-γ chain is expressed throughout the entire GI mucosa.

### Tissue mRNA Levels of FcεRIα do not Correlate with Serum IgE Levels

**Figure 5:**
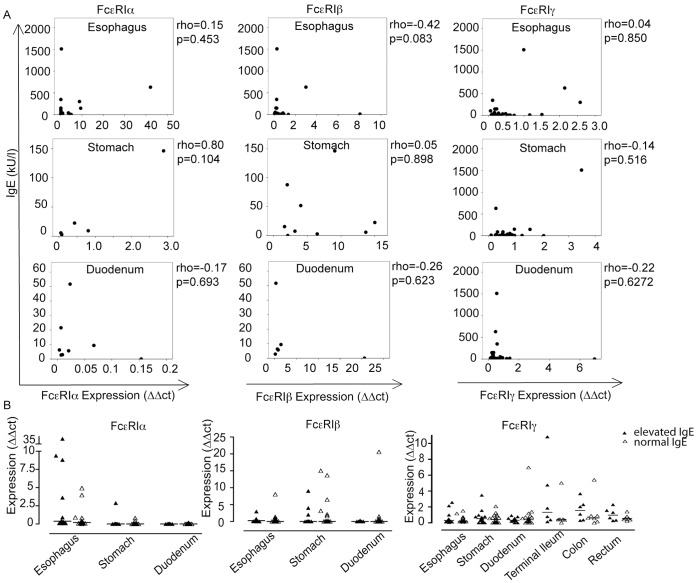
Correlation of FcεRI mRNA levels (ΔΔCt) and serum IgE levels (kU/l). (A) Scatter plots of serum IgE levels versus FcεRIα- (left column), FcεRIβ- (middle column), and FcεRIγ-mRNA transcripts (right column) in the esophagus (top row), the stomach (middle row), and the duodenum (bottom row). rho, Spearman rank correlation coefficient. (B) Comparison of mRNA levels of the three receptor subunits between patients with elevated IgE (high IgE, filled triangles) and patients with normal IgE (open triangles) did not reveal any significant difference (Mann-Whitney-U test).

IgE-binding to FcεRIα stabilizes the receptor complex at the cell surface [15]. It has also been suggested that IgE-binding promotes FcεRIα transcription [24]. Therefore, we investigated the correlation of serum IgE levels with tissue mRNA levels of the three FcεRI-subunits. In specimens from the esophagus, the stomach and the duodenum with detectable FcεRIα-, β-, or γ-mRNA expression, no correlations between serum IgE and any of the receptor subunits were found (Figure 5A, Spearman rank correlation). Additionally, a comparison of tissue mRNA levels of FcεRIα and β between children with elevated IgE and normal IgE did not show any significant differences in the upper GI tract (Figure 5B, left and middle panel). The highest number of FcεRIα mRNA transcripts was observed in the esophageal mucosa of patients with elevated IgE though (Figure 5B, left panel). FcεRIγ transcripts did not significantly differ between elevated- and normal IgE patients in the entire gut (Figure 5B, right panel, Mann-Whitney-U test, respectively). The low frequency of FcεRIα- and β-mRNA positive specimens did not allow for comparative analysis in the large intestine.

Our experiments permit us to conclude that our findings regarding the correlation of serum IgE levels and FcεRI expression in peripheral blood [13] cannot be extended to the GI mucosa.

### FcεRI mRNA Expression Levels are Modulated by Inflammation of the GI Tract

**Figure 6:**
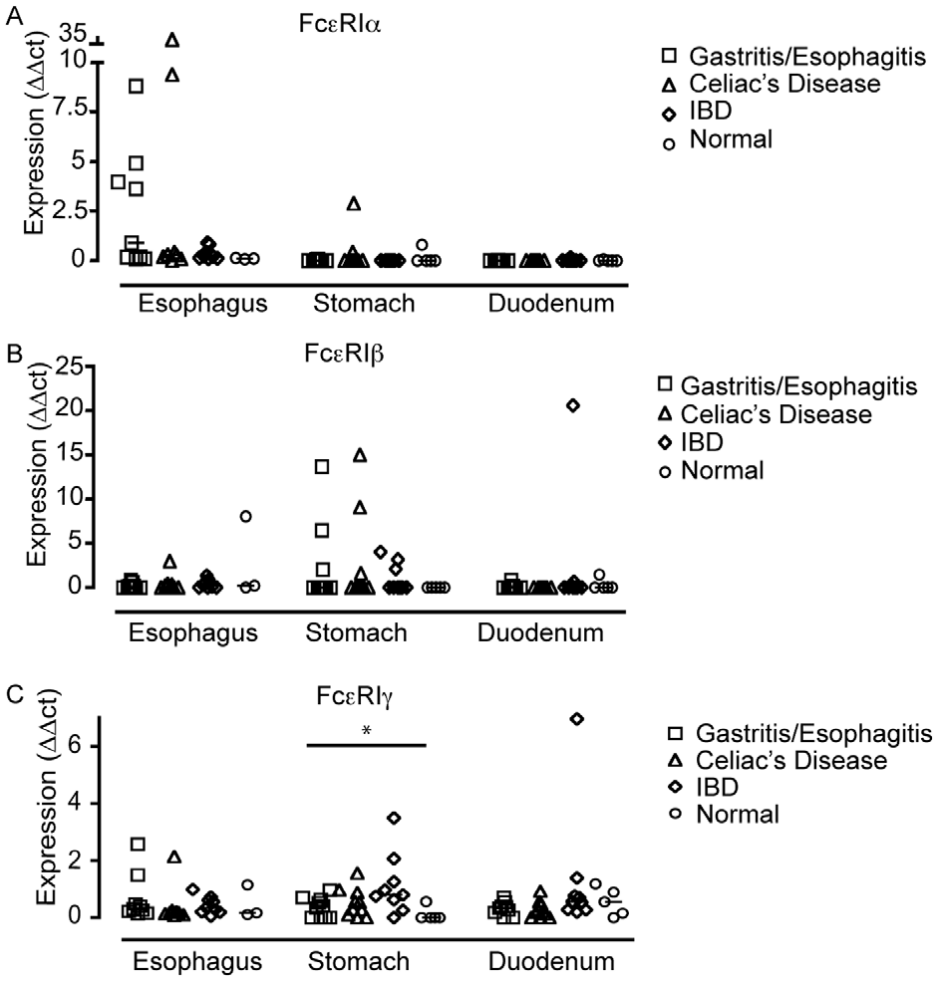
FcεRI mRNA expression levels in the upper gastrointestinal tract under inflammatory conditions. (A) FcεRIα-, (B) FcεRIβ-, and (C) FcεRIγ-mRNA expression levels in specimens from children with gastritis/esophagitis (open squares), celiac disease (open triangles), inflammatory bowel disease (IBD) (open diamonds), and normal mucosa (open circles). * p<0.05, Kruskal-Wallis test.

We have previously shown that FcεRI-bearing immune cells infiltrate the esophageal mucosa of children with reflux esophagitis and EoE, two inflammatory conditions of the esophagus [19]. We, therefore, hypothesized that the mucosa of patients with inflammatory conditions of the gut should express high levels of FcεRI transcripts due to the influx of inflammatory immune cells. In support of this hypothesis, we found that some gastritis/esophagitis patients in our study showed higher FcεRIα mRNA levels in the esophagus than celiac disease- and IBD-patients or normal controls (Figure 6A). We note that the specimens that contained FcεRIα mRNA in the terminal ileum, the colon, and the rectum were obtained from two IBD patients, while one colon biopsy from a normal control patient was also positive for FcεRIα mRNA. In the gastric mucosa, FcεRIβ mRNA levels appeared to be higher in diseased children than in normal controls (Figure 6B). These differences, however, were not statistically significant (measured using a Kruskal-Wallis test). A statistically significant difference between patient subgroups was found for the FcεRIγ expression levels in gastric specimens (Figure 6C, p = 0.031, Kruskal-Wallis test). Group-by-group comparison did not reveal statistically significant differences between the respective patient subgroups though (Mann-Whitney-U test with Bonferroni-Holm correction for multiple testing). In summary, our data suggest that inflammatory infiltration of the GI mucosa may be associated with an increase in FcεRI tissue mRNA expression, although the small patient cohort did not permit statistically significant results.

## Discussion

We show here that the high affinity IgE receptor, FcεRI, is highly expressed on cells infiltrating the mucosa of the esophagus, the stomach, and the duodenum, and shows a low level of expression in more distal parts of the GI tract. Accordingly, tissue mRNA of all three FcεRI subunits was detected in the intestinal mucosa. FcεRIα-, β-, and γ-mRNA levels did not correlate with total serum IgE, but were modulated by gastrointestinal inflammation.

FcεRIα mRNA was most abundant in the esophageal mucosa and could be detected in every patient. FcεRIβ mRNA levels, on the other hand, were rather low in the esophagus and peaked in the gastric mucosa. This could reflect the predominance of cells expressing the trimeric receptor isoform, such as dendritic cells and eosinophils, in the esophagus. In the stomach, cells bearing the tetrameric FcεRIαβγ2, such as mast cells and basophils, could be the main FcεRI-expressing population. This interpretation is supported by previous studies showing that the gastric mucosa is particularly rich in mast cells [25], [26], [27], whereas in the esophagus eosinophils and dendritic cells are the major FcεRI-positive populations [19]. The common Fc-γ-chain mRNA was abundantly found in almost all biopsies. The increasing levels of the Fcγ-chain along the GI system most likely reflect the physiologic increase in Fc-receptor-bearing mucosal immune cells even in healthy subjects [28], [29].

The binding of IgE, the natural ligand to FcεRI, increases FcεRI expression on peripheral blood cells and Langerhans cells [13], [30], [31] by stabilizing the receptor on the cell surface [15]. It has been suggested that IgE also upregulates FcεRIα transcription, although this hypothesis has been disputed [24], [32]. We did not find any significant correlation between mucosal FcεRIα-, -β-, or -γ-mRNA expression levels and serum IgE in any part of the GI tract. Our data thus support earlier observations that FcεRI expression is likely regulated on the protein rather than the mRNA level [32].

Total IgE values in mixed-age pediatric populations are not directly comparable due to differing age-related normal ranges. To overcome this problem, absolute IgE levels are commonly converted into age-normalized z-values as published by Kjellman et al. [33]. We did not detect a significant correlation when we used z-units instead of absolute IgE values either (data not shown). Nonetheless, it is possible that the mucosal immune system is more autonomous than previously assumed. Vicario et al. recently discovered local isotype class-switching to IgE and IgE production in the esophageal mucosa of EoE patients, regardless of their atopic status [34]. Local IgE production might also boost mucosal FcεRI-expression, independent of systemic serum IgE levels.

We observed that mucosal inflammation was associated with FcεRIα mRNA levels, based on our finding that patients with a histopathological diagnosis of esophagitis displayed the highest FcεRIα mRNA expression. The highest copy numbers of esophageal FcεRIα mRNA were found in two celiac disease patients. According to routine pathology records, these two patients also had intraepithelial inflammatory infiltrates in the esophagus. Therefore, high FcεRIα mRNA expression could be a surrogate for invading FcεRIα-positive inflammatory cells. Further supporting this argument, previous studies identified intraepithelial Langerhans cells and mast cells in the inflamed esophageal mucosa of children with EoE and reflux esophagitis as the main cellular sources of FcεRI [19], [35]. Other studies described the infiltration of IgE-loaded mast cells in the duodenal mucosa of patients with food-related diarrhea [21]. In line with these previous findings, we also identified intraepithelial mucosal mast cells and Langerhans cells as the FcεRI-expressing cells. With respect to the distal sections of the intestine, Untersmayr et al. recently identified epithelial FcεRIα expression in Paneth cells in small intestinal crypts and colon epithelium in colon cancer patients, and in patients with various gastrointestinal inflammatory conditions. Samples from healthy controls were negative for the receptor in this study. The authors, therefore, proposed a role for mucosal FcεRI expression in gastrointestinal pathology [20]. Our data support Untersmayr’s data as two out of nine IBD patients were positive for mucosal FcεRIα mRNA in the terminal ileum and the large intestine.

The role of the low affinity IgE receptor, CD23, in transepithelial transfer of IgE-allergen complexes is well established [5]. FcεRI, in contrast, does not seem to be involved in antigen-shuttling, as it is localized to infiltrating cells rather than epithelial cells. It is tempting to speculate that FcεRI may function as an antigen uptake-receptor on mucosal DCs with the capacity to sample allergen from the gastrointestinal lumen via FcεRI-bound specific IgE, as has been shown in mouse models [36], [37], [38].

In summary, we show here that FcεRI is robustly expressed in the upper GI mucosa of pediatric patients at steady state. Inflammatory conditions, independent of the underlying pathology, lead to increased FcεRI tissue mRNA expression. In conclusion, our data define the upper GI tract as the main site for IgE-mediated immune activation via FcεRI. Once the immune homeostasis is disturbed by an inflammatory condition such as IBD, the lower GI tract becomes susceptible to IgE-mediated immune reactions as well. The fact that we see tissue FcεRIα mRNA regulation by inflammatory conditions independent of IgE-mediated disorders indicates that this receptor might also play a role in pathologies other than allergies.

## Materials and Methods

### Ethics Statement

The study protocol was approved by the Ethic’s Committee of the Medical University of Vienna. Patients or their legal guardians provided written informed consent.

### Patients’ Tissue Specimens

Children between 2 and 18 years of age who underwent elective diagnostic endoscopy of the upper and/or lower GI tract at the Department of Paediatrics and Adolescent Medicine, Medical University of Vienna were randomly invited to participate. Subjects with increased risk of bleeding or perforation and patients scheduled for endoscopic interventions or undergoing non-elective endoscopy were excluded. Endoscopic biopsies from the esophagus, the stomach, the duodenal bulb, the terminal ileum, the ascending colon, and the rectum were either fixed in OCT Tissue Tek (Sakura Finetek, Staufen, Germany) and stored at −80°C until further processing for RT-PCR or snap-frozen for immunohistochemistry analysis. Tissue specimens were obtained from sites free of macroscopic mucosal pathology.

### Immunohistochemistry

FcεRIα in frozen sections of gastrointestinal specimens was detected with mAb 15.1 [31]. Sections were acetone-fixed, rehydrated and blocked in 2% (w/v) BSA/phosphate buffered saline (PBS). Endogenous peroxidases were quenched with 0.3% hydrogen peroxide/PBS for 15 minutes. Sections were exposed to blocking solution containing 2% horse serum for 20 minutes and incubated with mAb 15.1 or concentration-matched mouse IgG1 isotype control overnight at 4°C. Bound antibody was detected with Vectastain® ABC peroxidase system (Vector-labs, Peterborough, UK) according to the manufacturere’s protocol and visualized with AEC reagent (3-amino-9-ethylcarbazole; Dako, Carpinteria, CA, USA). Sections were counterstained with Papanicolaous Harris 1a stain (Merck, Darmstadt, Germany).

### Double Immunofluorescence Staining

Double immunofluorescence staining for FcεRIα and the mast cell marker c-kit or the Langerhans cell marker CD1a were performed on frozen esophagus specimens. Sections were acetone-fixed, incubated with 1 mg/ml sodium borohdyride (ICN Chemicals, Aurora, OH), and blocked with 5% v/v normal donkey serum (Jackson ImmunoResearch Lab Inc., West Grove, PA, USA). Sections were then exposed to mouse anti-FcεRIα (Cra 1, clone AER-37, 1∶100 dilution, eBioscience, San Diego, CA, USA) and goat anti-CD1a (clone N-19, 1∶200 dilution, Santa Cruz Biotechnology Inc., Santa Cruz, CA, USA) or rabbit anti-c-kit (A4502, 1∶250 dilution, Dako) overnight. CY5-conjugated donkey anti-mouse-, Dylight 549 donkey anti-goat-, and Dy549 donkey anti-rabbit-antibodies (Jackson ImmunoResearch Lab Inc., 1∶200 dilution) were used as secondary antibodies. Secondary antibody controls were performed in parallel with each experiment. After they were washed, the sections were mounted with Prolong Gold anti-fade mounting media containing DAPI (Invitrogen, Grand Island, NY, USA). FcεRIα/CD1a double-staining was investigated on a Zeiss AxioImager M1 wide-field fluorescent microscope with a 20x/0.8 N.A. plan-apochromat objective lens and the AxioVision software. FcεRIα/c-kit double staining was investigated on a Zeiss LSM510 Meta confocal system and Zeiss LSM510 image acquisition software. The images were taken with a 20x/0.8 plan-apochromat objective and a 40x/1.3 oil plan-apochromat objective.

### Real-time RT-PCR

Total mRNA was isolated from mucosal specimens using the GenElute™ Total RNA MiniPrep Kit (Sigma-Aldrich, St. Louis, MO, USA) and subjected to reverse transcription with the iScript™ cDNA synthesis kit (Bio-Rad, Hercules, CA, USA) following the manufacturer’s instructions. Quantification of mRNA was performed using the iQ SYBR Green Supermix (Bio-Rad) on a CFX96 Real time System (Bio-Rad). The thermal profile of the PCR reaction was: initial denaturation at 95°C for 3 minutes, followed by 30 cycles of denaturation at 95°C for 10 seconds and annealing/elongation at 63°C for 30 seconds, and a final melting curve. The following primers were used to amplify cDNA: FcεRI α: forward: 5′GAA ATG TGA CCT GCT GCT GA3′, reverse: 5′TGT GGC AGC TGG ACT ATG AG3′; beta-Actin: forward: 5′CTC TTC CAG CCT TCC TTC CT3′, reverse: 5′AGC ACT GTG TTG GCG TAC AG3′. Primer sets for FcεRIβ and -γ subunits and glyceraldehyde-3-phosphate-dehydrogenase (GAPDH) were purchased from Qiagen (Hilden, Germany). Relative expression of FcεRIα,-β and -γ was calculated according to the Pfaffl method [39] as follows: ΔΔCT = ΔCt(sample)- ΔCt(reference), where ΔCt(sample) is the Ct value of the gene of interest normalized to the endogenous housekeeping genes GAPDH and beta-Actin and ΔCt(reference) is the sample with the lowest ΔCt value. cDNA from palatine tonsils was used as positive control and included in each experiment as inter-run calibrator.

### Total Serum IgE Measurement

Serum samples for IgE measurement were collected at the time of endoscopy.

Total IgE was determined using the Phadia ImmunoCAP® solid phase immunoassay (Pharmacia Diagnostics, Uppsala, Sweden). Total serum IgE levels are given in kU/l. For some analyses, patients were subgrouped into elevated- and normal-IgE-patients. Expected normal ranges for this assay are 40 kU/l for age < one year, 100 kU/l for the 2^nd^ year of life, 150 kU/l for the 3^rd^ year of life, 190 kU/l for the 4^th^ and 5^th^ years of life, 150 kU/l for the 6^th^ year of life, 120 kU/l for the 7^th^ to the 16^th^ year of life, and 100 kU/l for patients older than 16 years.

### Statistical Analysis

Statistical analyses were carried out with SPSS software (Version 17.0 SPSS Inc., Chicago, IL, USA). Differences in tissue mRNA levels between groups were tested for statistical significance with two-sided Kruskal-Wallis-H- test and two-sided Mann-Whitney U-test with Bonferroni-Holm correction. Correlations of data with serum IgE were determined using Spearman rank correlation test. For certain analyses, total serum IgE values were transformed into z-units to adjust for age dependent normal values as published by Kjellman et al. [33]. P-values <0.05 were considered statistically significant.
